# Event-related and readiness potentials when preparing to approach and avoid alcohol cues following cue avoidance training in heavy drinkers

**DOI:** 10.1007/s00213-020-05462-7

**Published:** 2020-02-26

**Authors:** Lisa C. G. Di Lemma, Andrej Stancak, Vicente Soto, Nick Fallon, Matt Field

**Affiliations:** 1 UK Centre for Tobacco and Alcohol Studies, Liverpool, UK; 2 Faculty of Health and Social Care, University of Chester, Chester, UK; 3 Department of Psychological Sciences, University of Liverpool, Liverpool, UK; 4 Centre for Social and Cognitive Neuroscience, School of Psychology, Universidad Adolfo Ibáñez, Las Condes, Santiago, Chile; 5 Department of Psychology, University of Sheffield, Sheffield, UK

**Keywords:** Alcohol, Avoidance training, Cognitive bias modification, Event related potentials, Motor readiness potentials

## Abstract

**Rationale:**

Cue avoidance training (CAT) reduces alcohol consumption in the laboratory. However, the neural mechanisms that underlie the effects of this intervention are poorly understood.

**Objectives:**

The present study investigated the effects of a single session of CAT on event-related and readiness potentials during preparation of approach and avoidance movements to alcohol cues.

**Methods:**

Heavy drinking young adults (*N* = 60) were randomly assigned to complete either CAT or control training. After training, we recorded participants’ event-related and motor readiness potentials as they were preparing to respond.

**Results:**

In the CAT group, N200 amplitude was higher when preparing to approach rather than avoid alcohol pictures. In the control group, N200 amplitudes did not differ for approach and avoidance to alcohol pictures. Regarding the late positive potential (LPP), in the CAT group, the negativity of this was blunted when preparing to avoid alcohol pictures relative to when preparing to avoid control pictures. In the control group, the negativity of the LPP was blunted when preparing to approach alcohol pictures relative to when preparing to approach control pictures. There were no effects on motor readiness potentials. Behavioural effects indicated short-lived effects of training on reaction times during the training block that did not persist when participants were given time to prepare their motor response before executing it during the EEG testing block.

**Conclusions:**

After a single session of CAT, the enhanced N200 when approaching alcohol cues may indicate the engagement of executive control to overcome the associations learned during training. These findings clarify the neural mechanisms that may underlie the effects of CAT on drinking behaviour.

## Introduction

In alcohol consumers, alcohol-related cues evoke automatic approach tendencies, and these automatic tendencies are thought to influence drinking behaviour. A number of studies have measured the strength of these tendencies via the approach avoidance task (AAT; Wiers et al. 2009) and related tasks (Field et al. 2008) and demonstrated that non-dependent heavy drinkers, compared to light drinkers, are faster to approach alcohol pictures rather than avoid them (Kersbergen et al. 2015; Watson et al. 2012).

In alcohol-dependent patients, stronger automatic tendencies to approach alcohol are associated with activation in brain regions that underlie cue reactivity and craving (Schacht et al. 2013) such as the nucleus accumbens and medial prefrontal cortex (mPFC, Ernst et al. 2014; Wiers et al. 2014). Given the high spatial resolution of functional magnetic resonance imagining (fMRI) techniques, these studies help to clarify the neural architecture that underlies approach and avoidance tendencies in addiction. However, automatic approach and avoidance tendencies are activated within milliseconds of perceiving a salient stimulus, which means that fMRI lacks the temporal resolution to fully characterise the underlying brain mechanisms (Hajcak et al. 2010). A more complete understanding of the brain mechanisms that underlie approach and avoidance tendencies, including their temporal resolution, can be achieved by investigating event-related potentials (ERPs), and motor readiness potentials (Colebatch 2007), using electroencephalography (EEG), as participants complete these computerised tasks. Studies that employed cue exposure paradigms have demonstrated that the amplitude of the P300 and late positive potential (LPP) ERP components are significantly enhanced in substance users, relative to non-users, during exposure to substance-related cues (standardised mean difference (SMD) = 0.46 in Littel et al. 2012), whereas the N200 has been linked to executive control deficits in substance users (Petit et al. 2013; Smith et al. 2014). It is plausible that each of these ERP components is implicated in approach and avoidance tendencies that are evoked by substance-related cues.

Only a handful of EEG studies have investigated specific ERPs associated with automatic approach-avoidance tendencies in heavy drinkers and alcohol-dependent patients. Two studies measured ERPs in alcohol consumers as they prepared and executed a motor response during an alcohol AAT. These studies investigated these biases following administration of a small dose of alcohol (relative to a placebo), but both focussed on different aspects of EEG activity (desynchronisation of cortical oscillation and amplitude asymmetries), rather than ERPs. Both demonstrated that preparatory motor states seem to play a key role in performance on the AAT, by showing greater desynchronisation in the beta band cortical oscillation when preparing to approach alcohol following alcohol administration (Korucuoglu et al. 2014) and by observing greater preparatory lateralised beta activity when preparing to approach soft drinks, in heavy drinkers who were attempting to control their alcohol consumption (Korucuoglu et al. 2016).

More relevant to the focus of the present study are studies that used EEG to measure brain activity during an AAT with emotional stimuli. In one study, participants performed two blocks of an AAT, one that required emotion-congruent responses (i.e. approach positive pictures and avoid negative pictures) and another that required emotion-incongruent responses (i.e. approach negative and avoid positive; Ernst et al. 2013). The authors demonstrated increased amplitude of the N200, a marker of cognitive control and conflict resolution (Luijten et al. 2014; Clayson and Larson 2011; Folstein and VanPetten 2008), during emotion-incongruent compared to emotion-congruent trials. In a subsequent study (Bamford et al. 2015), the amplitude of the LPP, an ERP component associated with attentional visual processing of salient stimuli (Hajcak et al. 2010; Keil et al. 2002; Macnamara et al. 2009), was increased when participants were preparing to make an emotion-congruent response compared to an emotion-incongruent response. An earlier study demonstrated cortisol administration led to enhancement of P150 and P300 amplitudes before participants (who were high in threat sensitivity) made avoidance responses to angry facial expressions (Van Peer et al. 2007).

The research on approach tendencies evoked by substance-related cues has a clinical application in the form of cognitive bias modification (CBM), a group of computerised behaviour change interventions that have the common goal to train participants to overcome automatic approach tendencies and other cognitive biases, with a view to reducing alcohol consumption or other appetitive motivated behaviours such as food intake (Di Lemma and Field 2017; Gladwin et al. 2016; Kakoschke et al. 2017; Wiers et al. 2013). A specific form of CBM is cue avoidance training (CAT), based on the AAT in which participants categorise alcohol-related and control pictures by making approach and avoidance movements using a joystick. This intervention results in a reduction in the strength of automatic alcohol-approach associations such that alcohol cues evoke automatic avoidance responses when they are encountered in the future (Wiers et al. 2011). Importantly, compared to control interventions, CAT prompts reductions in alcohol consumption in the laboratory among non-dependent heavy drinkers (see Di Lemma and Field 2017; Sharbanee et al. 2014; Wiers et al. 2010) and it reduces the likelihood of relapse to drinking after treatment in alcohol-dependent patients (Wiers et al. 2011; Eberl et al. 2013; Manning et al. 2016; Rinck et al. 2018). Despite some consistent findings for CAT, there are concerns about the robustness and replicability of these findings (Cristea et al. 2016; Leeman et al. 2018; Wiers et al. 2018).

The psychological mechanisms that underpin the behavioural effects of CAT are fairly well-understood: the reversal of automatic approach bias (Eberl et al. 2013) and, in particular, the strengthening of automatic alcohol-avoidance associations (Gladwin et al. 2015) can account for the beneficial effects of CAT on long-term outcomes in alcohol-dependent patients. However, the brain mechanisms that underlie these changes in alcohol-avoidance and alcohol-approach associations after CAT have only recently been investigated, and they remain poorly understood (den Uyl et al. 2016a, b; Ferrari et al. 2018; Wiers et al. 2014; Wiers and Wiers 2016). Two fMRI studies demonstrated reduced activation in the amygdala (Wiers et al. 2015b) and in the mPFC (Wiers et al. 2015a) in alcohol-dependent patients after multiple sessions of CAT, which is suggestive of a blunting of activity in neural substrates of alcohol cue reactivity (Schacht et al. 2013).

To date, only one study has used EEG to investigate the brain mechanisms that underlie effects of CAT (den Uyl et al. 2016b), and this study found null effects on the P300 ERP component after a brief session of CAT. However, the main aim of the study was to investigate if transcranial direct current stimulation (tDCS) would enhance CBM effects, and EEG was recorded during an oddball cue-reactivity task rather than during preparation of motor activity during an approach/avoidance task. To address this gap in the literature, the purpose of the present study was to investigate changes in ERPs during preparation of motor activity that arise as a result of a single session of alcohol-CAT in non-dependent heavy drinkers.

In the present study, we measured participants’ EEG as they completed a modified version of an AAT with alcohol pictures, during a response-preparation period (preparatory AAT; Korucuoglu et al. 2014), immediately after they had been trained to associate alcohol with either avoidance or approach (CAT or control intervention). We investigated ERPs and motor readiness potentials (the contingent negative variation; CNV; Hillyard 1973; Rohrbaugh and Gaillard 1983; Walter et al. 1964), as critical precursors of the execution of goal directed behaviour that should be capable of detecting neural effects of associations learned (during CAT) on preparatory motor states, without being contaminated by motor activity. CNV is the most widely used EEG marker of motor preparation (the intention to perform an action) if the external cue occurs at a predictable time during a warning signal. CNV reflects a slow negative inflexion in EEG signals over frontal-central and parietal-central areas during the preparation period between a warning stimulus (S1) and an imperative stimulus (S2), usually beginning 1 s before the onset of motor activity, and which continues to rise until motor activity is initiated (Haggard 2019; Luck and Kappenman 2011).

In line with the findings on emotional stimuli, we predicted changes in manual reaction times, in amplitudes on a range of ERP components (P300, N200, LPP), and in the CNV, between the two groups of participants, when they were preparing to perform actions that were congruent with contingencies that were applied during the training phase, compared to actions that were incongruent with those learned during the training phase. Specifically, on the basis of previous studies (Bamford et al. 2015; Ernst et al. 2013; van Peer et al. 2007), we hypothesised that, in the ‘approach alcohol’ group, P300 and LPP amplitudes would be enhanced during preparation to approach alcohol stimuli, while an enhanced N200 should be observed when preparing to avoid alcohol stimuli. By contrast, in the ‘avoid alcohol’ group, we expected to see an enhancement of P300 and LPP amplitudes during preparation to avoid alcohol stimuli, alongside an enhanced N200 when preparing to approach alcohol stimuli. Additionally, we predicted similar congruency effects on readiness potentials, with greater CNV amplitudes on congruent trials in both groups during the preparation to respond to the AAT.

## Methods

### Participants

Sixty heavy drinkers (42 females, 18 males) were recruited from staff and students at the University of Liverpool via online and poster advertising. Inclusion criteria included average weekly alcohol consumption in excess of the UK Department of Health guidelines at the time of the study (at the time, these were 14 and 21 units per week for females and males respectively; note that these guidelines were revised in January 2016, after completion of this study). Participants were also required to be aged between 18 and 26, fluent in English, have normal or corrected to normal vision and no history of alcohol use disorders. We recruited young adult heavy drinkers in accordance with previous laboratory studies of CAT (e.g. Di Lemma and Field 2017; Korucuoglu et al. 2014; Wiers et al. 2011): heavy drinkers are well-represented in this population, in whom acute alcohol-related harm is problematic. The study was approved by the University of Liverpool Research Ethics Committee. All subjects gave written informed consent in accordance with the Declaration of Helsinki.

### Design

A between-subject design was employed. Participants were randomly allocated to one of two training groups, either avoid alcohol CAT (repeatedly avoiding alcohol pictures and approaching neutral pictures, 90–10% contingency) or approach alcohol (control group: reversed contingencies). The present control condition was employed instead of a ‘sham’ training condition (50% contingency), in order to increase the subjective value of the alcohol stimuli and inflate training effects (Schonberg et al. 2014), which should exaggerate differences between groups and therefore provide a more sensitive test of our hypotheses.

### Materials and tasks

Computer tasks were programmed and administered in Inquisit version 3.0 (Millisecond Software 2009) and were administered on a Dell desktop computer with a 15″ monitor. Participants responded using a joystick.

Twenty pairs of alcohol-related and matched neutral (control) pictures were used in the computer tasks (Barkby et al. 2012; Di Lemma and Field 2017; Field et al. 2004). Alcohol pictures depicted alcoholic drinks (e.g. bottles or glasses) and drinking scenes (e.g. models holding a beverage or drinking it), and each was matched to a neutral picture that depicted stationery (e.g. pens, staplers) and models using those items (e.g. holding pens or stapling paper).

During each trial, an alcohol-related or control picture was presented in the centre of the screen and participants were required to rapidly categorise pictures according to their spatial orientation (landscape or portrait), but to ignore the content of the pictures. Participants were instructed to ‘approach’ pictures presented in one format (e.g. portrait orientation) by pulling the joystick towards them and ‘avoid’ pictures presented in the other format (e.g. landscape orientation) by pushing the joystick away (making this an ‘irrelevant-feature’ AAT; see Kersbergen et al. 2015). During each trial, the picture remained on screen until the participant responded or until a 1000-ms timeout had elapsed. Correct approach responses caused a zooming effect (the picture became larger), and correct avoidance responses caused a shrinking effect (the picture became smaller). Incorrect responses or failure to respond in time led to error feedback in the form of a red cross displayed in the centre of the screen for 500 ms (see Di Lemma and Field 2017).

The experimental session comprised three blocks: (1) a pre-test AAT assessment block followed by (2) the main training block. Both the pre-test and training blocks were identical to those described in a recent study (Di Lemma and Field 2017). Immediately after the training block, participants completed (3) the preparatory AAT assessment block, which was based on that described in Korucuoglu et al. (2014). Participants were not informed when the task switched between assessment, training and preparatory blocks. Picture format was counterbalanced, with half of participants instructed to pull landscape and avoid portrait format pictures, and reversed instructions for the remaining participants. Participants were required to make an equal number of push and pull responses in all blocks. Trial order within each block was randomised.

#### Pre-test AAT assessment block (10 practice trials followed by 80 test trials)

This block contained 50% alcohol and 50% control pictures, half of each in portrait format and half in landscape format. In these blocks, participants had to approach and avoid alcohol and control pictures with equal frequency.

#### Training block (480 trials)

In this block (in which only a subset of 10 of the alcohol-related and 10 matched control pictures were used), for participants in the CAT group, alcohol pictures were presented in the format that required an avoidance response on 90% of trials and in the format that required an approach response on 10% of trials, whereas control pictures were presented in the format that required an approach response on 90% of trials and in the format that required an avoidance response on 10% of trials. These stimulus-response mappings were reversed in the control group. Participants were given a short break after 240 trials (see Di Lemma and Field 2017).

#### Pre-test Preparatory AAT (eight practice trials followed by 200 test trials interrupted with one block of 180 booster training trials; see Fig. 1)

**Fig. 1:**
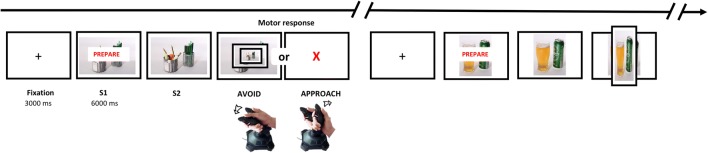
Schematic representation of the trial procedure during the post-training ‘preparatory’ AAT block

This block duplicated the 50:50 contingency between picture and movement type in the pre-test AAT assessment block (block 1, see above). In order to capture neural activity during preparatory motor states, the trial sequence in the post-test assessment block differed from that in other blocks (see Korucuoglu et al. 2014). On each trial, immediately after the fixation cross (3000 ms), the picture appeared on the screen with the word ‘PREPARE’ superimposed on top (3000 ms). During this preparation period, participants were asked to prepare their motor response (approach or avoid), depending on the feature of the stimulus (landscape or portrait orientation), but to withhold it until the word PREPARE disappeared. Any responses made during the preparation period were not registered. After 3000 ms, the ‘prepare’ text was removed, and participants were able to make their response. During each trial, the picture remained on screen until the participant responded or until a 1000 ms timeout had elapsed (see Fig. 1). Zooming effects for correct responses and error feedback for incorrect responses were identical to those applied during other blocks. This preparatory assessment AAT block was interrupted halfway (after 100 trials) by a single *booster training block* (180 trials), which was then followed by the remaining 100 post-test assessment trials. This was done in order to ensure that the repeated performance of a 50:50 contingency, during this assessment block, would not undermine the associations learned during training. Additionally, due to the long procedure and the requirement for prolonged attention and motionlessness, participants were offered a break after every 25 trials.

#### EEG recordings

EEG activity was recorded during the preparatory AAT block (block 3, see above). EEG activity was recorded continuously using 64 channels (scalp electrodes) based on the extended 10/20 system using a Biosemi ActiveTwo system (Biosemi, Amsterdam, the Netherlands). The electrode cap was aligned using four anatomical landmarks: nasion, occipital protuberance and left and right pre-auricular points (Chatrian et al. 1985; Klem et al. 1999; Jasper 1958). Electrode gel was used to ensure that electrode to skin impedance was kept below 10 kΩ. Vertical electrooculograms (EOG) were recorded in parallel with EEG signals above and below the right eye using flat disc electrodes, and all signals were recorded continuously with 1024 Hz sampling frequency. The recording bandpass filter was set at 0.1–512 Hz. Data was spatially transformed to reference-free data using the common average reference method (Lehmann 1984).

#### Procedure (see Fig. 2)

Participants were tested between 12:00 and 18:30 in the EEG laboratory on the University of Liverpool campus, in a single experimental session that lasted no more than 2 h. Participants provided informed consent and a breathalyser reading (all participants had a breath alcohol content of zero), before being seated at a desk approximately 1.5 m away from the computer monitor. After providing informed consent, electrodes were fitted and tested before participants completed the three blocks of the AAT as described above. Finally, the EEG cap and electrodes were removed before participants provided general demographic information and completed three questionnaires: the Timeline Follow-Back retrospective alcohol diary (TLFB; Sobell and Sobell 1992), the Alcohol Use Disorders Identification Test (AUDIT; Saunders and Babor 1993) and the Readiness to Change Questionnaire (RTCQ; Rollnick et al. 1992). At the end of the experiment, participants were debriefed and compensated either with course credits or shopping vouchers (£20 Sterling).

**Fig. 2:**
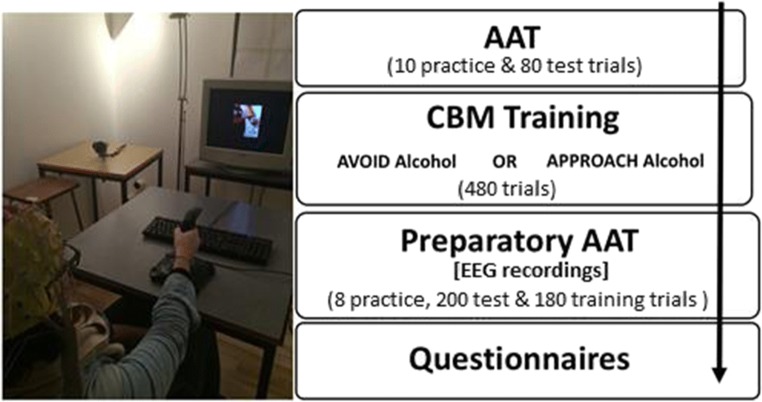
Schematic overview of the experimental procedure. For details, see ‘Methods’

#### Behavioural data reduction and analysis

In order to analyse behavioural data (latencies to approach and avoid alcohol and control pictures) during the pre-test and post-test assessment blocks, and over time during the training block, we first excluded trials with errors and those with outlying reaction times. Two separate outlier cut-offs were computed: one for the pre-training and training blocks and another for the post-training block (in which reaction times were affected by the introduction of the preparatory phase at the start of each trial). RTs faster than 200 ms or slower than 2000 ms, then those that were more than three SDs above the mean for that block were excluded. After excluding trials with errors and outliers in this way, RTs were analysed using mixed design ANOVAs as detailed below.

#### EEG data reduction and analysis

Brain Electrical Source Analysis v.6.0 program (BESA, GmbH, Germany; Scherg and Berg 1990) was used for pre-processing of EEG data during the preparatory phase of each trial in the post-test block. EOG artefacts were removed by a principal component analysis procedure (Berg and Scherg 1994), and muscle artefact rejection was completed manually by visual inspection before averaging. CNV (Tecce 1972; Luck and Kappenman 2011) was used to investigate continuous EEG data, during the remaining epochs of 3000 ms from the preparation phase of the trials, with ERPs time-locked from the onset of the picture that appeared on the screen with the word PREPARE superimposed (S1) until when the word PREPARE disappeared from the screen which signalled to participants that they could make their response (S2; see Fig. 1). These epochs were averaged across all trials of the post-assessment block, for each condition (approach alcohol, avoid alcohol, approach control, avoid control). Filtering was performed on the averaged data at 0.01–40 Hz. For individual electrode analysis, grand averages were computed in BESA and exported to EEGLab v10.2.5.8b (Delorme and Makeig 2004) for Matlab R2009a (Mathworks: Natick, MA). Then, identification and analysis of ERPs (associated with the processing of the stimuli) and of the CNV (related to the readiness potential of preparatory motor action) was guided by visual inspection of the waveforms. This led to the identification of three peak ERP amplitudes (P100, N200 and LPP). However, contrary to expectations, the P300 ERP was not detected. For these ERP components, five clusters of electrodes were detected and ERP amplitude data were analysed using repeated measures ANOVAs in SPSS v.22 (IBM Inc., USA). A similar cluster analysis was also conducted on four 500 ms intervals, from 1000 to 3000 ms, on the CNV to examine training effects on preparatory motor actions.

## Results

### Group characteristics (Table 1)

Table 1 shows summary data for the self-report measures separately for groups (2: avoidance training, approach training). A MANOVA showed that groups were not well matched (*F* (7, 52) = 2.53, *p* = .03). There were significant between group differences in age (*F* (1, 58) = 6.68, *p* = .01; participants in the approach training group were younger) and AUDIT scores (*F* (1, 58) = 7.16, *p* = .01; participants in the approach training group had higher scores). No other differences were observed for weekly alcohol consumption and readiness to change (RTCQ); (*F*s < 1.38, *p*s > .25). A chi-square test confirmed that groups were well-matched for gender ratio (*χ*^2^(1) = 1.27, *p* = .26).

**Table 1:** Participant characteristics by group. Values are mean (± SD)

	Avoidance training group	Approach training group	MANOVA *F* value
Age (years)	26.77 (5.12)	23.67 (4.11)	*F* = 6.68, *p* = .01
Gender ratio (M/F)	11:19	7:23	N/A
Weekly alcohol consumption	24.40 (10.90)	22.49 (12.93)	*F* = 0.38, *p* = .54
AUDIT	10.10 (4.29)	14.10 (6.09)	*F* = 7.16, *p* = .01
RTCQ pre-contemplation	− 1.00 (3.82)	− 1.17 (2.93)	*F* = 0.04, *p* = .85
RTCQ Contemplation	0.27 (3.32)	0.93 (3.50)	*F* = 0.57, *p* = .45
RTCQ Action	− 0.37 (4.33)	− 1.63 (4.02)	*F* = 1.38, *p* = .25

Weekly alcohol consumption = self-reported typical weekly alcohol intake, in UK units

AUDIT = Alcohol Use Disorders Identification Test, values range from 0 to 40

RTCQ = Readiness to Change Questionnaire subscales range from − 8 to + 8

### Behavioural data (Table 2)

#### Pre-test assessment block

Reaction times to initiate approach and avoidance movements were subjected to a 2 × 2 × 2 mixed design ANOVA, with within-subject factors of Picture type (2: alcohol, control) and Movement (2: approach, avoidance) and a between-subject factor of Group (2: avoidance training, approach training). The main effect of Movement was statistically significant (*F* (1, 58) = 7.01, *p* = .01), reflecting faster RTs to initiate approach rather than avoidance movements. The hypothesised two-way interaction Picture type × Movement (*F* (1, 58) = .08, *p* = .79) was not significant, and there were no other significant main effects or interactions (*F*s < 2.37, *p*s > .13).

**Table 2:** Reaction times (milliseconds) to approach and avoid alcohol and control pictures during the approach-avoidance task (AAT), the post-training assessment task (preparatory AAT) and at the beginning and end of the training blocks. Values are mean (± SD), between-group contrasts are independent samples *t* tests

	Avoidance training group	Approach training group	Between group contrasts
AAT (Pre-CAT)			
Approach alcohol	748.96 (143.32)	765.14 (159.44)	*t =* − 0.41 (*p* = .*68*)
Avoid alcohol	794.91 (173.07)	772.24 (138.36)	*t =* 0.56 (*p* = .*58*)
Approach control	751.07 (143.71)	753.03 (141.71)	*t =* − 0.05 (*p* = .*96*)
Avoid control	783.19 (167.43)	766.57 (142.79)	*t =* 0.41 (*p* = .*68*)
CAT (during training blocks)			
Beginning—approach alcohol	763.00 (120.89)	729.92 (129.96)	*t =* 1.02 (*p* = .*31*)
Beginning—avoid alcohol	759.65 (152.37)	808.47 (167.91)	*t =* − 1.18 (*p* = .*24*)
Beginning—approach control	735.52 (116.63)	765.07 (137.76)	*t =* − 0.90 (*p* = .*37*)
Beginning—avoid control	770.25 (147.24)	745.22 (148.33)	*t =* 0.66 (*p* = .*51*)
End—approach alcohol	744.39 (139.96)	735.73 (163.46)	*t =* 0.22 (*p* = .*83*)
End—avoid alcohol	734.89 (135.02)	792.12 (177.41)	*t =* − 1.41 (*p* = .*16*)
End—approach control	725.33 (116.58)	774.79 (139.42)	*t =* − 1.49 (*p* = .*14*)
End—avoid control	804.15 (165.03)	718.25 (165.64)	*t =* 2.01 (*p* = .*05*)
Preparatory AAT (Post-CAT)			
Approach alcohol	580.02 (132.27)	572.29 (167.69)	*t =* 0.20 (*p* = .*84*)
Avoid alcohol	600.96 (148.55)	582.05 (132.68)	*t =* 0.52 (*p* = .*61*)
Approach control	582.34 (146.16)	567.17 (154.77)	*t =* 0.39 (*p* = .*70*)
Avoid control	596.33 (157.45)	585.59 (137.53)	*t =* 0.28 (*p* = .*78*)

Post-hoc exploratory planned contrasts for the sample as a whole showed that participants were in general faster to initiate approach movements rather than avoidance movements to both alcohol pictures (*M* = 757.05, SD = 150.52 vs. *M* = 783.58, SD = 155.75, *t* (59) = − 2.14, *p* = .04, *d* = .17) and control pictures (*M* = 752.05, SD = 141.58 vs. *M* = 774.88, SD = 154.50, *t* (59) = − 2.13, *p* = .04, *d* = .15). Latencies to approach (*t* (59) = .58, *p* = .57) and avoid (*t* (59) = − 1.03, *p* = .31) alcohol and control pictures did not differ. Therefore, contrary to expectations, participants did not possess an automatic tendency to approach rather than avoid alcohol pictures (relative to control pictures) during the pre-test block.

#### Training block

In order to explore the formation of cue-response associations over the course of the training block, RTs to initiate approach and avoidance movements were subjected to a 2 × 2 × 2 × 2 mixed design ANOVA, with within-subject factors of Time (2: first eight trials of each type at the beginning of the training block vs. the last eight trials of each type at the end of the training block), Picture type (2: alcohol, control) and Movement (2: approach, avoidance) and a between-subject factor of Group (2: avoidance training, approach training). Again, the main effect of Movement was statistically significant (*F* (1, 58) = 5.58, *p* = .02), RTs to initiate approach were generally faster than RTs to initiate avoidance movements. Additionally, the three-way interaction Picture type × Movement × Group was significant (*F* (1, 58) = 19.25, *p* < .01), and the two-way interaction Picture type × Group (*F* (1, 58) = 3.88, *p* = .06) and the three-way interaction Movement × Time × Group both approached significance (*F* (1, 58) = 3.22, *p* = .08). Importantly, the critical four-way interaction Time × Picture type × Movement × Group was not significant (*F* (1, 58) = 1.44, *p* = .24) and there were no other significant main effects or interactions (*F*s < 2.52, *p*s > .12).

Given the significant three-way interaction that was not qualified by time, data were averaged across the beginning and end of the training block. Planned contrasts separately for each group revealed that participants in the avoidance training group were faster to avoid alcohol pictures (*M* = 747.27, SD = 132.15) compared to control pictures (*M* = 787.20, SD = 147.36), *t* (29) = − 3.01, *p* = .01, *d* = .29, but latencies to approach alcohol and control pictures did not differ (*t* (29) = 1.75, *p* = .09). By contrast, participants in the approach training group were faster to approach alcohol pictures (*M* = 732.82, SD = 134.54) compared to control pictures (*M* = 769.93, SD = 129.04; *t* (29) = − 2.11, *p* = .04, *d* = .28), and they were also faster to avoid control pictures (*M* = 731.73, SD = 147.88) compared to alcohol pictures (*M* = 800.29, SD = 165.47; *t* (29) = 3.81, *p* < .01, *d* = .44).

#### Post-test assessment block

RTs to initiate approach and avoidance movements immediately after the preparatory phase of each trial were subjected to a 2 × 2 × 2 mixed design ANOVA, with within-subject factors of Picture type (2: alcohol, control) and Movement (2: approach, avoidance) and a between-subject factor of Group (2: avoidance training, approach training). Again, the main effect of Movement approached significance (*F* (1, 58) = 3.43, *p* = .07), reflecting a general speeding of approach relative to avoidance movements. The expected three-way interaction Picture type × Movement × Training condition was not significant (*F* (1, 58) = 1.01, *p* = .32), and there were no other significant main effects or interactions (*F*s < .09, *p*s > .76).

Post-hoc planned contrasts, split by training group showed that participants in the avoidance training group, were faster to approach rather than avoid alcohol pictures (*M* = 580.20, SD = 132.27 vs. *M* = 600.96, SD = 148.55); *t* (29) = − 2.03, *p* = .05, *d* = .15. None of the other contrasts (e.g. approach alcohol vs. approach control; approach control. vs avoid control) in the avoidance training group were statistically significant (*t*s < 1.12, *p*s > .27). In the approach training group, none of the contrasts were statistically significant (*t*s < 1.24, *p*s > .23).

### ERP components and readiness potentials

**Fig. 3:**
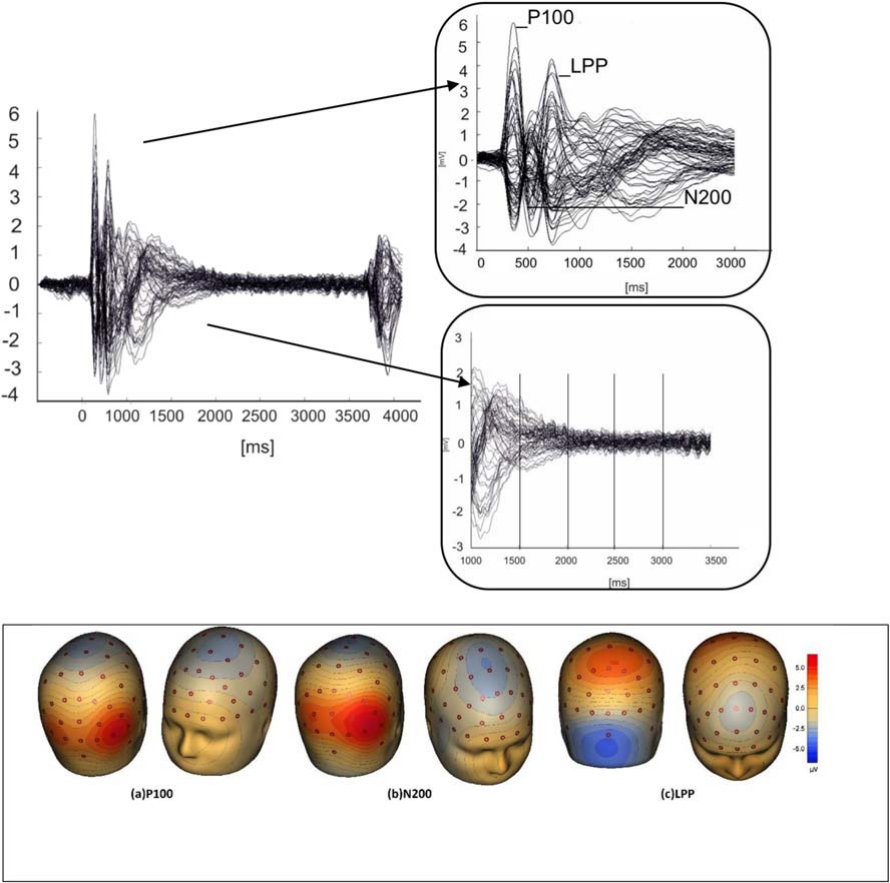
On the top left is the butterfly plot of grand average ERP responses and readiness potential to alcohol and control stimuli during the preparatory phase and corresponding scalp topographies. In the two panels on the top right, we highlight peak latencies of the distinct ERP components (123–143, 261–281 and 570–610 ms) and the following four 500 ms intervals of the readiness potential (CNV) to preparatory states to motor actions are shown. Underneath, the topographic maps of grand average ERPs overlaid on the volume rendering of the human head are shown below. **a** Latency component peaking at 123 ms (P100). **b** Latency component peaking at 261 ms (N200). **c** Latency component peaking at 570 ms (LPP)

ERPs in response to alcohol and control stimuli across all trials are illustrated in the form of a butterfly plot and topographic maps of the selected components (see Fig. 3). The grand average ERPs indicate that the topography across recording sites was generally consistent with that reported by other studies (Brunia and van Boxtel 2001; Tecce 1972).

The first component peaked at around 123 ms and showed positivity in the occipital electrodes and negativity over frontal electrodes, which is consistent with characteristics of the P100 component that is implicated in early visual processing (Hopf et al. 2002; Heinze and Mangun 1995; Maurage et al. 2012). This component is best represented by the first positive peak following presentation of the first stimulus (S1) on electrodes P07 and P08, which were analysed together as a first cluster.

The second component, which peaked at around 261 ms, showed strong central cortical negativity and parietal positivity which is consistent with characteristics of the N200 (Patel and Azzam 2005). This component is best represented as the first negative peak occurring after P100 on electrodes Fz and Cz, and these were analysed together as a second cluster.

The third component peaked at around 570 ms in the parietal (Pz, P2, P1) and mid-line electrodes (Fz, Cz), with strong negativity over the central occipital sites (Fz, Cz) and positivity over the central parietal sites (Pz, P2 and P1). This component is consistent with characteristics of the late positive potential (LPP).

Contrary to hypotheses, the anticipated P300 was not observed; consequently, it was not reported or analysed. Inconsistent observations of the P300 have also been found in studies that investigated responses to emotional stimuli during performance of the AAT (Bamford et al. 2015; Ernst et al. 2013).

Additionally, the plot evidenced no changes in electrophysiological activity before the stimulus that indicated that participants should perform the motor response (S2). However, exploratory analyses were conducted on the CNV in steps of 500 ms in the mid-line electrodes (Fz and Cz cluster) for four intervals starting from 1000 to 3000 ms, in order to confirm (as observed from the plot) null effects on the readiness potentials.

#### P100 (Fig. 4)

P100 amplitudes (averaged across electrodes P07 and P08) were subjected to a 2 × 2 × 2 mixed design ANOVA, with within-subject factors of Picture type (2: alcohol, control) and Movement (2: approach, avoidance) and a between-subject factor of Group (2: avoidance training, approach training). The main effect of Movement was statistically significant (*F* (1, 58) = 7.49, *p* = .01), reflecting a stronger peak in the P100 to initiate approach rather than avoidance movements. However, the three-way interaction Picture type × Movement × Training condition was not observed (*F* (1, 58) = .16, *p* = .69) and there were no other significant main effects or interactions (*F*s < 2.09, *p*s > .15).

**Fig. 4:**
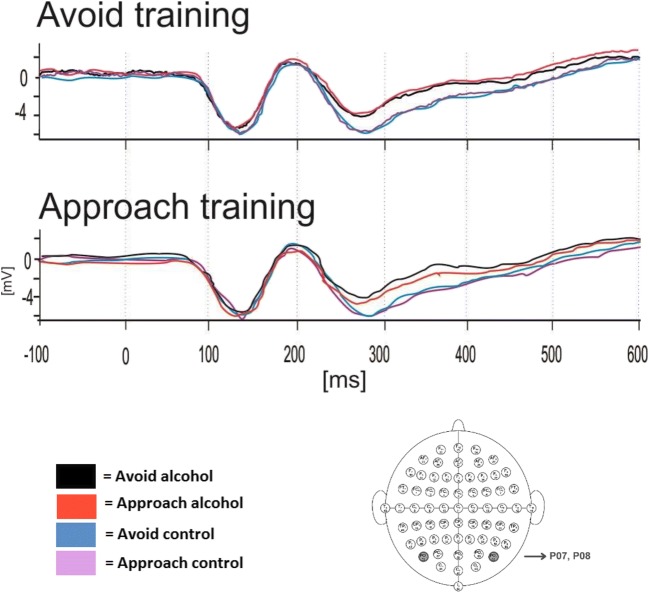
Grand average of ERP responses to alcohol and control stimuli during the preparation to respond to the AAT. Latency component 123 ms (P100) at parietal (P07 and P08) electrode sites as shown below by the 64-channel sensor net layout

#### N200 (Fig. 5)

A similar ANOVA was conducted to explore the influence of cue avoidance training on N200 amplitudes (averaged across electrodes Fz and Cz). A significant main effect of Picture type (*F* (1, 58) = 37.65, *p* < .01) was subsumed under the hypothesised three-way interaction Picture type × Movement × Group (*F* (1, 58) = 8.74, *p* = .01). There were no other significant main effects or interactions (*F*s < 1.41, *p*s > .24).

**Fig. 5:**
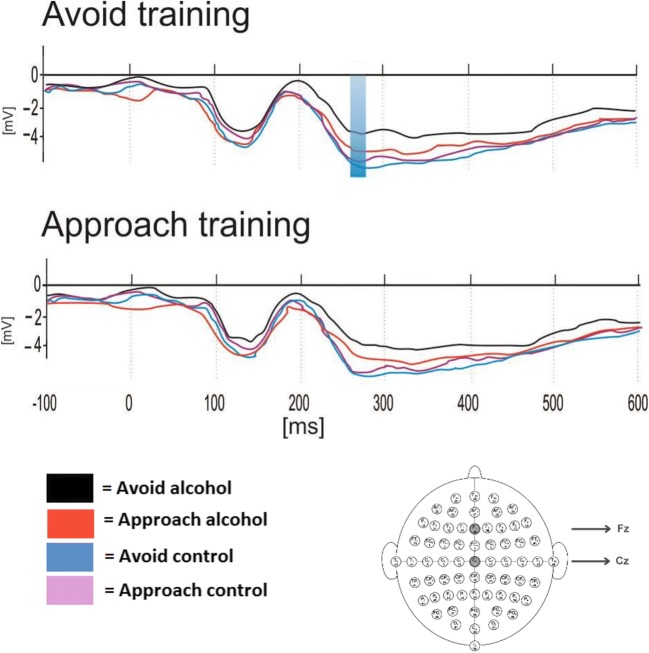
Grand average ERP responses to alcohol and control stimuli during the preparation to respond to the AAT. Latency component 261 ms (N200) at midline (Fz, Cz) electrode sites as shown below by the 64-channel sensor net layout

Post-hoc *t* tests performed separately on each group demonstrated greater negativity for control pictures relative to alcohol pictures in both groups of participants. More importantly, greater negativity in the N200 was seen in the avoidance training group when they were preparing to approach alcohol pictures compared to when preparing to avoid those pictures (*t* (29) = 2.34, *p* = .03, *d* = .24). However, N200 amplitudes to control pictures did not differ during preparation of approach and avoidance in this group (*t* (29) = − 1.11, *p* = .28). In the approach training group, N200 amplitudes when preparing to approach vs. avoid did not differ for either type of picture (alcohol: *t* (29) = − 1.24, *p* = .23; control: *t* (29) = 1.06, *p* = .30).

#### LPP (Fig. 6)

The influence of cue avoidance training on the LPP at parietal (Pz, P2 and P1) and midline (Fz, Cz) electrode sites was investigated with a 2 × 2 × 2 × 2 mixed design ANOVA, with within-subject factors of Picture type (2: alcohol, control), Movement (2: approach, avoidance) and Electrode site (parietal, midline) and a between-subject factor of Group (2: avoidance training, approach training). A significant main effect of Electrode site (*F* (1, 58) = 79.73, *p* < .01) and a two-way interaction Electrode site × Picture type interaction (*F* (1, 58) = 5.26, *p* = .03) were subsumed under the hypothesised four-way interaction Picture type × Movement × Electrode site × Group, which approached significance (*F* (1, 5 8) = 3.82, *p* = .06). There were no other significant main effects or interactions (*F*s < 2.94, *p*s > .09).

**Fig. 6:**
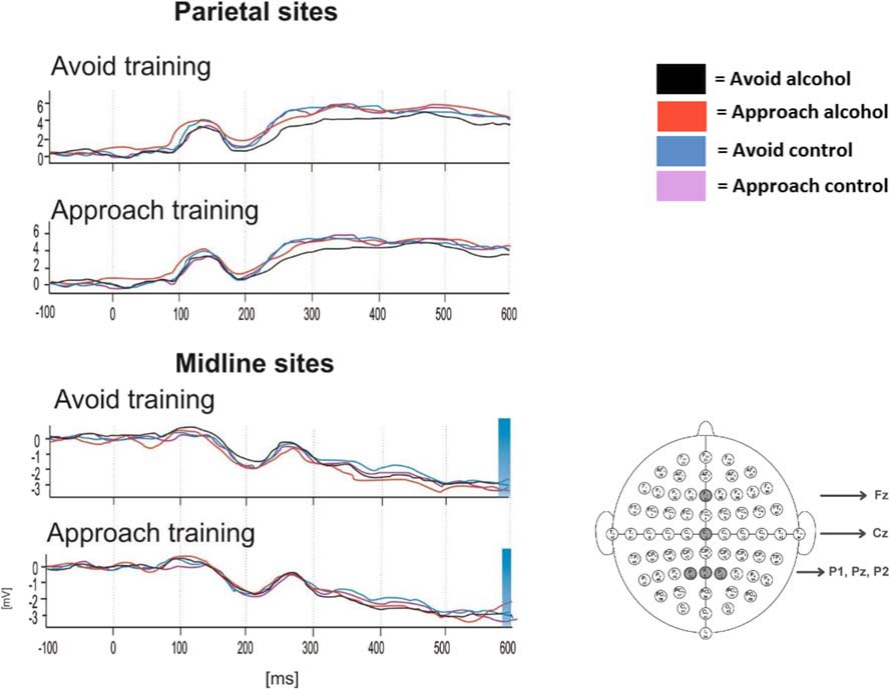
Grand average ERP responses to alcohol and control stimuli during the preparation to respond to the AAT. Latency component 570 ms (LPP) at parietal (Pz, P1 and P2) and midline (Fz, Cz) electrode sites as shown below by the 64-channel sensor net layout

Separate ANOVAs on LPP amplitudes at each electrode site confirmed that group differences were driven by the midline electrodes, which evidenced a statistically significant three-way Picture type × Movement × Group interaction (*F* (1, 58) = 4.41, *p* = .04). Data from the parietal electrodes revealed no significant main effects or interactions (*F*s < .44, *p*s > .51).

Post-hoc *t* tests were performed on LPP amplitudes at the midline electrodes. In the CAT group, LPP negativity was blunted when preparing to avoid alcohol pictures (*M* = − 3.23, SD = 7.04) relative to control pictures (*M* = − 4.41, SD = 6.33); *t* (29) = − 2.90, *p* = .01, *d* = .18. No other differences were observed in this group (*t*s < 1.77, *p*s > .09). By contrast, the reverse pattern was seen in the approach training group, in whom LPP negativity was blunted when preparing to approach alcohol pictures (*M* = − 1.82, SD = 3.98) relative to control pictures (*M* = − 2.75, SD = 3.10); *t* (29) = 2.17, *p* = .04, *d* = .26. There were no other significant differences in this group (*t*s < .97, *p*s > .34).

#### Preparatory readiness potential intervals in the mid-line electrodes (Fig. 7)

The amplitudes at the midline electrodes (Fz, Cz) were explored in four 500 ms intervals over time (1000–3000 ms). We hypothesised greater readiness potential on trials that were congruent with motor responses learned during the training block, which would indicate preparation for motor activity in line with the contingencies applied. However, observations from the plot showed no readiness potential (a clear negative shift prior S2). In order to validate these observed findings, we conducted a 2 × 2 × 2 × 2 mixed design ANOVA, with within-subject factors of Picture type (2: alcohol, control), Movement (2: approach, avoidance) and Time (1000 ms, 1500 ms, 2000 ms, 2500 ms) and a between-subject factor of Training condition (2: avoidance training, approach training). Results showed no significant change over time between groups, as the critical four-way interaction was not significant (*F* (3,174) = .03, *p* = .99). Only a main effect of picture type was found (*F* (1, 58) = 4.17, *p* = .05), indicating less motor readiness for alcohol pictures (*M* = − 14.83, SD = 51.63), relative to control pictures (*M* = − 19.38, SD = 47.45), *t* (29) = 2.05, *p* = .05, *d* = .65. No other main effects or interactions were observed (*F*s < 3.00, *p*s > .09), confirming observations from the butterfly plot.

**Fig. 7:**
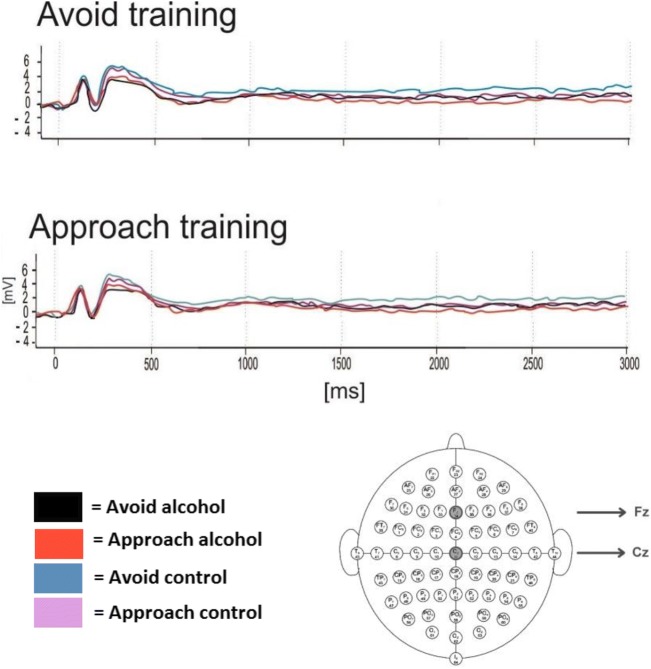
Grand average preparatory readiness potential (CNV) to approach and avoidance responses to alcohol and control stimuli during the preparation to respond to the AAT. Four 500 ms intervals at midline (Fz, Cz) electrode sites as shown below by the 64-channel sensor net layout

## Discussion

The primary novel finding from the present study was the demonstration of stronger negativity of the N200 ERP component in the CAT group when they were preparing to execute the motor movement that was incongruent with the alcohol-avoidance associations that they had learned during the training block. Comparable incongruency effects were not seen in the approach alcohol (control) group. However, in both groups of participants, blunted negativity of the LPP was observed at midline electrodes when participants were preparing to respond to alcohol-related pictures with a motor movement that was congruent with that which they had learned during the training block, i.e. blunted LPP negativity in the avoid alcohol group when preparing to avoid alcohol pictures, but blunted LPP negativity in the approach alcohol (control) group when preparing to approach alcohol pictures. Contrary to expectations, no changes in preparatory readiness potentials were observed in either group.

As expected, a greater N200 amplitude was observed in the CAT group when they were preparing to approach alcohol cues rather than avoid those cues. Our interpretation is that, after participants have repeatedly practised avoiding alcohol-related pictures and therefore formed associations between alcohol and avoidance, when they are subsequently required to approach alcohol-related pictures, this creates a response conflict that requires engagement of executive functions in order to resolve. This interpretation of the N200 findings is consistent with other studies that suggest that enhanced N200 is an important ERP marker of the engagement of executive control in heavy drinkers. For example, a study showed larger amplitude of the N200 in heavy drinkers when they were actively inhibiting a motor response during a Go–No Go task (Kreusch et al. 2014). Findings are also consistent with a prior AAT study with emotional stimuli which showed enhanced N200 amplitudes during incongruent trials (e.g. when preparing to avoid rather than approach positive stimuli; Ernst et al. 2013). This interpretation is also supported by demonstrations of increased engagement of inhibitory control towards high calorie foods, measured by larger N200 and P300 amplitudes, relative to low calorie foods (Carbine et al. 2018).

Contrary to expectations and findings from previous studies (e.g. Carbine et al. 2018; Littel et al. 2012), we did not detect enhanced P300 during exposure to alcohol-related pictures compared to control pictures. However, these findings are consistent with those from other studies that also used the alcohol AAT (den Uyl et al. 2016b) and the emotional AAT which failed to observe changes in the P300 (Bamford et al. 2015; Ernst et al. 2012). Importantly, we did observe the hypothesised congruency effects in the LPP. The amplitude of this component at midline electrodes was blunted when participants were preparing motor movements to alcohol stimuli that were congruent with associations learned the training block, and this was seen in both the CAT and the approach alcohol (control) group. These effects are in line with our predictions of emotion-congruency effects in this EEG component, as previously reported in an AAT study with emotional stimuli (Bamford et al. 2015). However, independently of training effects, we did not observe a potentiation of the LPP in response to alcohol versus control images in our study (i.e. the main effect of picture type was not statistically significant in the present study). This is inconsistent with findings from a meta-analysis which demonstrated robust increases in LPP amplitude when substance users viewed substance-related cues relative to control cues (Littel et al. 2012).

Regarding behavioural results, in line with some literature (Di Lemma and Field 2017; Manning et al. 2016; Wiers et al. 2015b), we did not observe robust increases in the strength of alcohol-avoidance associations in participants who completed a single session of CAT. During the pre-training assessment block, the entire sample demonstrated a general bias to initiate approach rather than avoidance movements to all pictures, a pattern that has been observed in previous studies (Kersbergen et al. 2015; Watson et al. 2012). In line with previous literature, during the training block, learning effects were detected in the expected direction in both groups (Wiers et al. 2010, 2011; Eberl et al. 2013, 2014; Sharbanee et al. 2014; Gladwin et al. 2015). During the post-assessment block, in which participants had the opportunity to prepare their motor response before initiating it, these motor speeding effects reverted back to an overall approach bias in the CAT group. This suggests that effects of CAT on approach and avoidance response times are very sensitive to experimental factors (see Ferrari et al. 2018), in the present case because the training effects disappear (and are actually reversed) if a delay is imposed between participants planning their response and actually initiating it. This issue may also be exacerbated by the methodological limitations of the irrelevant-feature AAT task (assessment version: poor internal reliability and predictive validity) which may render it relatively insensitive for the purposes of assessing changes in alcohol approach-avoidance associations that are expected to arise after CAT (see Kersbergen et al. 2015).

This study has some limitations. The use of a ‘preparatory AAT’ (Korucuoglu et al. 2014, 2016) appears to have blunted some of the stimulus associations learned during the training block, which may have suppressed readiness potentials. This suggests that if participants are forced to wait before responding, they can quite easily resolve the conflict and reinstate the dominant motor response (which is to approach alcohol, i.e. approach bias). This is in line with a recent Inhibitory Control Training (ICT; another form of CBM intervention; see Jones et al. 2016, 2018) study which suggested that time pressure is essential in order to observe training effects (Veling et al. 2017): if there is no time pressure on responding, the effects of ICT on reaction times disappear. Thus, the clinical implication is that any beneficial effects of CAT on behaviour might be completely eliminated if participants have the opportunity to stop for a moment and think after they have been exposed to an alcohol-related stimulus. Additionally, the control condition used in the present study (alcohol approach training) is suitable for investigating basic mechanisms because it is likely to accentuate between-group differences. However, this control condition is not translatable to clinical investigations of CAT and other forms of CBM. Future laboratory studies could compare the effects of CAT with a more neutral control condition such as those used in trials with clinical populations (e.g. Eberl et al. 2013).

A further limitation is that preparatory readiness potentials were not observed in either group. This may be related to the measure or task adopted. We adopted a CNV paradigm because our paradigm had fixed (and therefore predictable) stimulus onsets. Other studies have shown the absence of readiness potentials prior to conscious actions triggered by unpredictable external stimulus (Haggard 2019; Haggard and Clark 2003; Libet et al. 1993), yet the time may not have been that predictable to participants, especially due to a long and varied session of tasks. Future studies should consider task timings and alternative ways to trigger the readiness potentials. Our failure to observe the hypothesised training effects on reaction times and some of the EEG measures could be attributed to the fact that we administered only a single, relatively brief training session, whereas most clinical studies of CAT have administered multiple sessions (Eberl et al. 2014). Future studies should consider if longer and/or multiple sessions of CAT have more robust effects on behavioural and EEG measures. Although all participants had a breath alcohol content of zero, future studies could measure the duration of participants’ abstinence from alcohol before the experimental session in order to investigate if this is associated with behavioural or EEG measures. Finally, we excluded participants with an alcohol use disorder from taking part in the present study because it would have been unethical to expose such participants to the control (sham training) intervention that could have increased their motivation to drink alcohol. Future studies should attempt to replicate our findings in alcohol-dependent populations by incorporating a neutral comparison condition that is suitable for a dependent population.

Most importantly, this is the first EEG study to explore event-related and readiness potentials following a single session of CAT in a sample of heavy drinkers. Thus, the present findings are an important proof of concept of the mechanisms underpinning CAT, which are necessary in order to optimise these training interventions and apply them in real-world settings and clinical populations (see Cristea et al. 2016).

To conclude, we demonstrated that a brief session of CAT yielded behavioural learning effects only during training blocks and generated changes in neural activity when participants were preparing to respond to alcohol-related cues by making an approach or avoidance response. CAT resulted in increasing N200 amplitude when preparing to approach alcohol cues, which suggests recruitment and engagement of executive control when participants have to approach alcohol pictures immediately after having been trained to avoid those pictures. Additionally, in all participants, the negativity of the LPP was blunted when participants were preparing to make a motor movement (approach or avoid alcohol, depending on the contingencies applied during training) that they had repeatedly practised during the training block.
